# Patterns and management of degloving injuries: a single national level 1 trauma center experience

**DOI:** 10.1186/s13017-016-0093-2

**Published:** 2016-07-27

**Authors:** Suhail Hakim, Khalid Ahmed, Ayman El-Menyar, Gaby Jabbour, Ruben Peralta, Syed Nabir, Ahammed Mekkodathil, Husham Abdelrahman, Ammar Al-Hassani, Hassan Al-Thani

**Affiliations:** 1 Trauma Surgery Section, Hamad General Hospital, Doha, Qatar; 2 Clinical Research, Trauma Surgery Section, Hamad General Hospital, Doha, Qatar; 3 Clinical Medicine, Weill Cornell Medical College, Doha, Qatar; 4 Department of Radiology, Hamad General Hospital, Doha, Qatar

**Keywords:** Degloving, Soft tissue injury, Debridement, Management, Trauma

## Abstract

**Background:**

Degloving soft tissue injuries (DSTIs) are serious surgical conditions. We aimed to evaluate the pattern, management and outcome of DSTIs in a single institute.

**Methods:**

A retrospective analysis was performed for patients admitted with DSTIs from 2011to 2013. Presentation, management and outcomes were analyzed according to the type of DSTI.

**Results:**

Of 178 DSTI patients, 91 % were males with a mean age of 30.5 ± 12.8. Three-quarter of cases was due to traffic–related injuries. Eighty percent of open DSTI cases were identified. Primary debridement and closure (62.9 %) was the frequent intervention used. Intermediate closed drainage under ultrasound guidance was performed in 7 patients; however, recurrence occurred in 4 patients who underwent closed serial drainage for recollection and ended with a proper debridement with or without vacuum assisted closure (VAC). Closed DSTIs were mainly seen in the lower extremity and back region and initially treated with conservative management as compared to open DSTIs. Infection and skin necrosis were reported in 9 cases only. Open DSTIs were more likely involving head and neck region and being treated by primary debridement/suturing and serial debridement/washout with or without VAC. All-cause DSTI mortality was 9 % that was higher in the closed DSTIs (19.4 vs 6.3 %; *p* = 0.01).

**Conclusion:**

The incidence of DSTIs is 4 % among trauma admissions over 3 years, with a greater predilection to males and young population. DSTIs are mostly underestimated particularly in the closed type that are usually missed at the initial presentation and associated with poor outcomes. Treatment guidelines are not well established and therefore further studies are warranted.

## Background

Degloving soft-tissue injuries (DSTIs) are often serious surgical conditions characterized by avulsions or detachment of the skin and subcutaneous tissue from the underlying muscle and fascia secondary to a sudden shearing force applied to the skin surface [1]. DSTIs are more commonly observed in males due to disproportionately higher burden of traumatic injuries [2]. Although it may occur anywhere in the body, the main sites of DSTIs are lower extremities, trunk, scalp and face with a variable amount of skin and soft tissue loss [3–5]. DSTIs could be categorized as either closed/internal or open/external lesions [6]. Delayed diagnosis and treatment of these injuries often result in full-thickness necrosis due to jeopardized blood supply to the avulsed skin flap [7]. Moreover, patients with severe DSTI could develop infection or even necrotizing fasciitis due to mismanagement leading to high morbidity and mortality [6].

DSTIs are mostly underestimated lesions due to lack of clinical diagnostic and prognostic indicators and well established treatment guidelines. In addition, the marked variability in the type, magnitude and severity of DSTI makes it difficult to set a standard management and to predict outcomes. In closed or internal degloving injury the shearing forces create a cavity which subsequently gets filled with hematoma and liquefied fat [8]. Such closed internal degloving lesions usually develop over the greater trochanter and are known as Morel-Lavallee lesions [9].

The DSTI treatment varies considerably from close observation to active interventions such as early primary definitive skin closure, superior skin cover, early return of function and secondary procedures, if needed [5]. However, distinction between viable and nonviable tissues may be difficult during early wound management in both types of injuries [6]. Since, every injury is unique with variety of lesions; it is difficult to develop an appropriate decision-making algorithm for treatment and therefore, the outcome of DSTI often remains underestimated. Interestingly, there is a paucity of information on DSTI from our region in the Arab Middle East. In this study, we retrospectively reviewed the frequency, pattern, management and outcome of DSTIs from a single institute over a 3-year period in Qatar.

## Methods

Data were acquired retrospectively for all DSTI patients identified from the trauma registry database who were admitted to the section of trauma surgery at Hamad general hospital (HGH) between January 2011 and November 2013. HGH is the only Level 1 trauma center facility in Qatar which admits and treats all traumatic injury patients. DSTIs are defined as avulsion of soft tissue, in which an extensive portion of skin and subcutaneous tissue is detached from the underlying fascia and muscles [6]. We mainly diagnosed DSTI by clinical assessment, ultrasound and CT scanning. DSTI are classified as either open or closed. In an open DSTI, the skin is torn off the body though it may still be attached as a flap. Closed DSTI are soft tissue injuries with disintegration of the underlying layers in which the subcutaneous tissue is torn away from the underlying fascia, creating a cavity filled with hematoma and liquefied fat (i.e., Morel-Lavallée lesion). We excluded patients in whom the skin is completely detached as these are considered open wounds rather than DSTIs. Patients with open wounds are more likely to require some form of advanced soft tissue coverage. On arrival, all patients underwent thorough clinical assessment and resuscitation according to Advanced TRAUMA Life Support (ATLS) guidelines. Collected data included age, gender, mechanism of injury, injury severity score (ISS), type of degloving injury (open and closed), anatomical location (head, neck, back, limbs, abdomen and perineum), associated injuries, comorbidities, laboratory findings, blood transfusion and management [primary debridement/suturing; initial conservative treatment; serial debridement and washout with or without vacuum assisted closure (VAC)]. The term (early) vs (late) was used based on the initial treatment after the initial assessment. Complications (infection, skin necrosis and flap necrosis), discharge disposition (plastic surgery or rehabilitation), length of stay and mortality were also reported. Baseline demographic characteristics, mechanism of injury, site of injury, associated injury, management, and outcomes were also compared according to open and closed type of DSTIs.

### Statistical analysis

Data were expressed as proportions, medians, or mean ± standard deviation (SD), as appropriate. Differences in categorical variables between respective comparison groups were analyzed using Chi-Square test or Fisher exact (observed cell values less than 5) test for categorical variables. The continuous variables were analyzed using student’s *t*-test. Two-tailed p values < 0.05 were considered to be significant. Multivariate analysis was performed to look for ISS whether it has a prognostic role or not in both types of DSTIs. Data analysis was carried out using the Statistical Package for Social Sciences version 18 (SPSS Inc. Chicago, Illinois, USA).

## Results

**Fig. 1:**
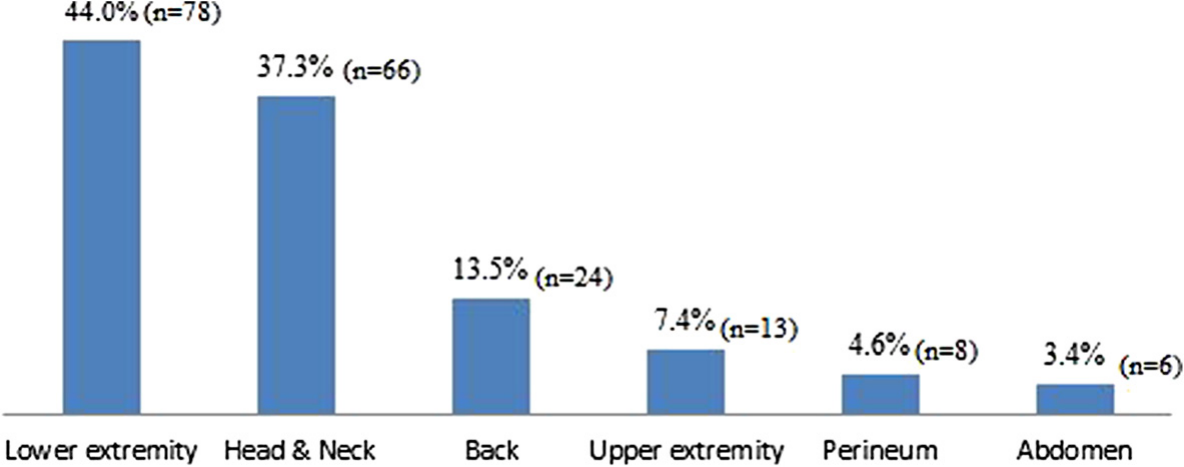
Distribution of anatomic site of degloving soft tissue injuries (DSTI)

A total of 178 patients with DSTIs were included in this study who was admitted to the Section of Trauma Surgery during three years period. The mean age of patients was 30.5 ± 12.8 years, and the majority were males (91 %) and expatriates (83.3 %). Demographics, clinical presentation, laboratory findings and type of DSTI are presented in Table 1. Motor vehicle crash (MVC) was the leading mechanism of injury (54.5 %) followed by falls from height (12.9 %) and pedestrian injuries (12.4 %). Lower extremity (40.4 %), head (23.0 %), upper extremity (19.1 %) and pelvis (16.9 %) were the most frequent associated injuries. Co-morbidities included diabetes (3.4 %) and hypertension (1.7 %). The median myoglobin level was 846 with a range from 21 to 6698 ng/ml. A higher proportion of cases sustained open/external type (79.8 %) DSTI followed by closed/internal type (20.2 %). The most frequent anatomic site of DSTI was lower extremity (44 %) followed by head/neck (37.3 %) and back (13.5 %) region (Fig. 1).

**Table 1:** Demographics and patient characteristics by type of degloving injury

	Overall (*n* = 178)	Open (*n* = 142)	Close (*n* = 36)	*P*
Age; years (mean ± SD)	30.5 ± 12.8	30.2 ± 13.1	31.9 ± 11.9	0.51
Males	162 (91 %)	129 (91.5)	33 (91.7)	0.97
Nationality				
Qatari	28 (16.7 %)	25 (18.3)	3 (9.7)	0.08 for all
Non-nationals	140 (83.3 %)	112 (81.7)	28 (90.3)	
Mechanism of Injury				0.63 for all
Motor vehicle crashes	97 (54.5 %)	80 (56.3)	17 (47.2)	
Fall from height	23 (12.9 %)	16 (11.3)	7 (19.4)	
Pedestrian injuries	22 (12.4 %)	16 (11.3)	6 (16.7)	
Fall of heavy objects	16 (9.0 %)	13 (9.2)	3 (8.3)	
Others	20 (11.2)	17 (12 %)	3 (8 %)	
Associated injuries				
Head	41 (23 %)	36 (25.4)	5 (13.9)	0.15
Lower extremity	72 (40.4 %)	58 (40.8)	14 (38.9)	0.83
Upper extremity	34 (19 %)	28 (19.7)	6 (16.7)	0.67
Pelvic fracture	30 (17 %)	19 (13.4)	11 (30.6)	0.01
Spinal	25 (14.2 %)	21 (15.0)	4 (11.1)	0.55
Solid organ injury	14 (7.9 %)	9 (6.3)	5 (13.9)	0.13
Facial	14 (8 %)	14 (9.9)	0.0	0.05
Chest	10 (5.7 %)	7 (5.0)	3 (8.3)	0.44
Bowel	6 (3.4 %)	3 (2.1)	3 (8.3)	0.09
Injury severity score (mean ± SD)	13.80 ± 10.9	13.11 ± 10.2	16.5 ± 13.04	0.09
Degloving injury size^a^ (*n* = 37)	90 (18–1080)^b^	75 (18–741)	380 (36–1080)	0.003
Anatomic site of DSTI^c^				
Head/Neck	66 (37.3 %)	65 (46.1)	1 (2.8)	0.001
Lower extremity	78 (44 %)	55 (39.0)	23 (63.9)	0.007
Upper extremity	13 (7.4 %)	12 (8.6)	1 (2.9)	0.24
Back	24 (13.5 %)	12 (8.5)	12 (33.3)	0.001
Perineum	8 (4.6 %)	6 (4.3)	2 (5.7)	0.72
Abdomen	6 (3.6 %)	4 (2.8)	2 (5.6 %)	0.41

^a^CT volume in cc, ^b^data present as median and range, ^c^there are overlapped sites

**Fig. 2:**
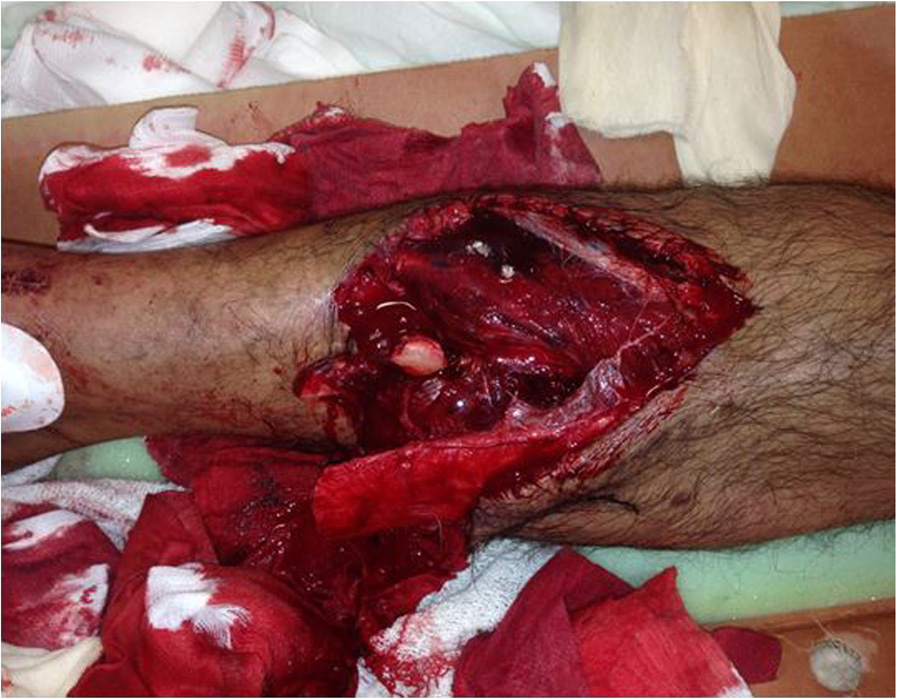
Open degloving with flap and underlying bony injury

**Fig. 3:**
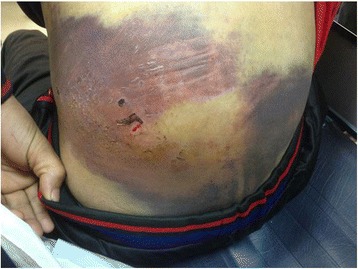
Closed degloving with frank bruising but intact skin

Table 2 shows the laboratory results, management, complications and outcome according to the type of degloving injury. Figures 2 and 3 show examples of open and closed degloving injuries.

**Table 2:** Management, complications and outcome by type of degloving injury

	Overall (*n* = 178)	Open (*n* = 142)	Close (*n* = 36)	*P*
Laboratory results				
Myoglobin	1190 ± 1179^b^	1076 ± 1125	1636 ± 1293	0.02
Blood Urea Nitrogen	4.8 ± 1.6^b^	4.6 ± 1.4	5.5 ± 2	0.004
Serum Creatinine	86.6 ± 37.3^b^	81.6 ± 31	107 ± 52	0.01
White blood cells	17.6 ± 7.7^b^	18 ± 8	16 ± 7	0.09
Platelets	250.8 ± 86.5^b^	258 ± 86	224 ± 84	0.04
Hemoglobin	12.9 ± 2.6^b^	13 ± 2.5	12.6 ± 3	0.42
INR	1.2 ± 0.3^b^	1.19 ± 0.3	1.19 ± 0.2	0.92
Serum Lactate	3.9 ± 2.4^b^	4.05 ± 2.6	3.31 ± 1.9	0.72
Treatment of degloving Injury				
Packed RBC units	3 (1–25)^a^	2 (1–20)	6 (1–25)	0.03
Fresh Frozen Plasma units	6 (2–24)^a^	5.5 (2–24)	6 (4–13)	0.46
Platelets units	9 (1–28)^a^	10 (1–28)	5 (1–11)	0.19
Primary Debridement/suturing	112 (62.9 %)	105 (73.9 %)	7 (19.4 %)	0.001
Initial conservative	28 (15.7 %)	0 (0.0 %)	28 (77.8 %)	0.001
Serial debridement^b^	34 (19.1 %)	33 (23.2 %)	1 (2.8 %)	0.003
Late flap	22 (12.3 %)	21 (14.8 %)	1 (2.8 %)	0.05
Disposition				
Plastic surgery	26 (14.6 %)	23 (16.4 %)	3 (8.3 %)	0.22
Rehabilitation	7 (3.9 %)	7 (4.9 %)	0 (0.0 %)	0.17
Hospital length of stay; days	10 (1–393)^a^	11 (1–393)	6 (1–365)	0.11
Complications				
Skin Infection	7 (3.9 %)	6 (4.3 %)	1 (2.8 %)	0.68
Skin necrosis	2 (1.1 %)	2 (1.4 %)	0 (0.0 %)	0.47
Mortality	16 (9.0 %)	9 (6.3 %)	7 (19.4 %)	0.01

^a^Median and range, ^b^Serial debridement and washout (with or without vacuum assisted closure)

The blood transfusion was needed primarily for associated injuries, rather than the degloving injury per se. Primary debridement and closure (62.9 %) was the main intervention for DSTI cases followed by serial debridement and washout with or without VAC (19.1 %). Intermediate closed drainage was done under ultrasound guidance for 7 cases out of which recurrence observed in four cases that had to undergo closed serial drainage for recollection. The definitive treatment for these patients was finally a proper debridement with or without VAC. One patient had undergone serial drainage over a period of three months before final resolution. Initial conservative management was adopted in 28 (15.7 %) patients. Late flap (i.e., serial debridement that eventually required rotational skin flap to close the defect) coverage was needed in 22 (12.3 %) cases. In those who underwent serial debridement some of them required closure by secondary suturing and others left to heal by secondary intention. Complications such as infection and skin necrosis were observed in 3.9 % and 1.1 % cases, respectively. Plastic surgical referral was sought for 26 (14.6 %) patients. The median hospital length of stay was 10 (1–393) days.

There were no differences between the two groups with respect to age, gender, mechanism of injuries and associated injuries. However, the frequency of pelvic fracture was significantly higher in the closed group (30.6 % vs. 13.4 %; *p* = 0.01) as compared to open DSTI. Regarding the anatomic site, DSTI at the lower extremity (63.9 % vs. 39 %, *p* = 0.007) and back (33.3 % vs. 8.5 %, *p* = 0.001) region were significantly higher in closed group compared to open group. In contrast, head (scalp) and neck region were mainly affected in the open group of degloving injuries (46.1 % vs. 2.8 %, *p* = 0.001) than closed group.

In comparison to closed DSTI, patients in open group were more likely to be treated by primary debridement/suturing (73.9 % vs. 19.4 %, *p* = 0.001) and serial debridement and washout with or without VAC (23.2 % vs. 2.8 %, *p* = 0.003). On the other hand, the frequency of initial conservative management (77.8 % vs. 0 %, *p* = 0.001) was higher in patients with closed group when compared to open DSTI. There was no significant difference in terms of complications or discharge dispositions between the two groups. The overall mortality was 9.0 % (*n* = 16) and around half (*n* = 7) of them died within the first 24 h of admission due to severe associated injuries. Moreover, the mortality rate was significantly higher in the closed group (19.4 % vs. 6.3 %, *p* = 0.01) as compared to open DSTI group. The mean ISS was greater in the closed DSTI in comparison to the opened type of DSTI (16.5 ± 13.04 vs 13.11 ± 10.2; *p* = 0.09). Multivariate analysis showed that ISS is a predictor of mortality in closed DSTI (Odd ratio 1.2; 95 % confidence interval 1.06-1.35; *p* = 0.004), however, this effect on mortality was not observed in the opened type of DSTI (Odd ratio 1.07; 95 % confidence interval 0.99-1.45; *p* = 0.07).

## Discussion

This is a unique study from the Arab Middle Eastern region which provides an insight on the frequency, patterns, management and outcome of DSTIs among trauma patients in Qatar. This is a large single-institution study which enrolled 178 patients as compared to earlier descriptive studies [10–12]. Our study shows that the incidence of DSTI is around 4 % with a greater predilection to males and young patients. Three quarter of the cases is traffic-related injuries. It has significant implications for the treatment and final outcome of our trauma patients. Most of the current literature on DSTI is mainly based on specific anatomic regions and are usually drived from case series or case reports. An earlier study from South Africa reviewed 16 cases with closed degloving injuries treated during one-year period [10]. Another study from Pakistan demonstrated the pattern of degloving injuries in 50 cases; of which majority sustained open type of degloving injuries [11]. Consistent with small number of cases, Milcheski et al. [12] reported 21 patients with degloving injuries from Brazil. In the present study, majority of the DSTI patients were young males which reflect the disproportionately higher burden of road traffic injuries in Qatar. Our findings are consistent with previous reports, which also documented a higher involvement of young males (88 %) in road traffic injuries [13].

DSTIs are often associated with severe concomitant injuries and massive blood loss [6].

Early diagnosis of DSTI remains challenging as the initial clinical evaluation could not predict avulsion of underlying soft tissue particularly in the closed DSTI [14]. On the other hand, prompt recognition of these injuries are crucial as treatment may be time consuming and such delay may increase the risk of infection or progression to necrotizing fasciitis. Severity of DSTI mainly depends on the mechanism of injury, comorbidities (particularly Diabetes mellitus), concomitant injuries, anatomic site and type (open or closed) of DSTI [6]. Our study showed MVC to be the most common cause of DSTI with frequent involvement of lower limb and head/neck regions. Consistent with our findings, several studies have demonstrated a higher association of lower limb DSTI and MVC [10, 12]. Similarly, Khan et al. [11] reported that higher frequency of young males (74 %) had degloving injuries of the lower limb. The present study also showed greater frequency of open DSTI which mainly affect head (scalp) and neck region. Although, less frequent closed DSTI were mainly associated with lower extremity and back. Contrarily, an earlier study reported greater involvement of open type (94 %) in patients with degloving injuries of the lower limb [11]. The present analysis showed that ISS was greater in the closed DSTI in comparison to the opened type, moreover ISS was found as a predictor of mortality only in the closed type of DSTIs.

**Fig. 4:**
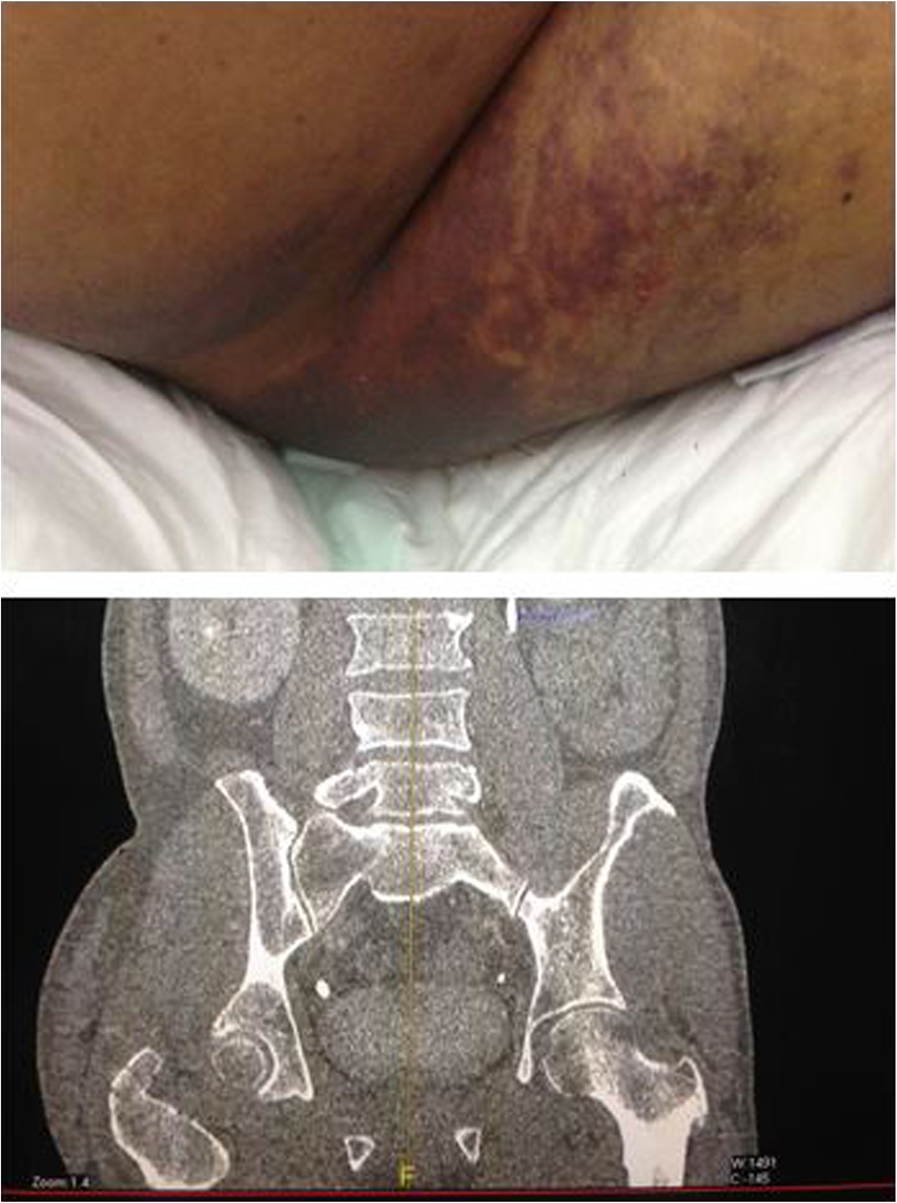
Morel Lavallee lesion (common site) and Coronal CT view of the lesion

Diagnosis of DSTI can be made by clinical assessment of fluctuant area as well as using imaging modalities such as ultrasonography, computed tomography (Fig. 4) and magnetic resonance imaging (MRI) [14]. Open DSTI is clinically self-evident condition that usually presented as a soft tissue loss of variable extent together with avulsed skin, subcutaneous tissue flaps from the underlying deep tissues which is the hallmark of physical finding together with overlying abrasion, ecchymosis or skin wound [9]. However, the diagnosis of closed DSTI is usually difficult and can be missed on the initial clinical evaluation and require radiological investigation for accurate diagnosis. Closed degloving injury with suspected Morel-Lavallée lesions (Fig. 4) could be diagnosed by CT scan, however, evaluation using MRI is more informative [15]. As ultrasound typically shows these lesions as anechoic or hypoechoic, with or without echogenic foci or even fluid/fluid levels. Therefore, for such cases MRI is the modality of choice which clearly determines the relationship of the collection with the underlying fascia [9, 14].

Direct injury to the cutaneous layers may result in necrosis of the skin overlying the degloved area. It can also occur on a delayed basis secondary to swelling of the degloved cavity, resulting in ischemia of the overlying skin [16]. To prevent potential complications such as, secondary infection and necrosis, early diagnosis and intervention are needed. In our series, skin necrosis was developed only in two cases with open DSTI and was not evident in the closed type of injuries. Although, skin necrosis was commonly considered as a complication of closed degloving injury, it was not observed in any of the patients in our series.

The primary management approach for DSTI ranges from optimal preservation of individual structure to early primary definitive skin cover, superior skin cover, early return of function and secondary procedures, if required [5]. Particularly, various modalities for the treatment of open DSTI include simple debridement with repair to more complex procedures like flaps, skin grafts, free tissue transfer, replantation or revascularization depending on the site, extent, severity and availability of the treatment. In our series, nearly 74 % of the cases with the open type and 20 % of the closed type of DSTI underwent primary debridement and closure. Plastic surgery consultation was sought in 26 (23 open and 3 closed) DSTIs cases due to wound complexity which necessitated a complex wound management including flap coverage and skin reconstruction.

Vacuum-assisted closure is an advanced management therapy often used to cover open degloving wounds of the lower-limb [17–19]. Utility of this device to develop the wound bed for grafting gained wide applicability which is directly applied to the wound to promote granulation tissue formation and skin grafting [18]. The present study showed that thirty four patients who needed serial debridement and washout due to re-accumulation were benefitted with an early wound closure from VAC therapy.

Management of closed DSTI is more challenging due to lack of evidence-based guidelines, these injuries are either treated by non-operative therapy or percutaneous and operative techniques. In our study, majority of patients with closed DSTI (78 %) underwent conservative treatment. Hak et al [9] performed open debridement of the Morel-Lavallée lesion with the incision placed close to the middle of the degloved area with thorough exploration for possible loculations. However, due to high incidence of complications such as re-accumulation of hematoma, wound breakdown and infection, the authors left the wound open post debridement [9]. A retrospective study by Nickerson et al [20] reported various treatment options for Morel-Lavallée lesions or closed DSTI such as compression wraps or observation, percutaneous aspiration or operative management with incision/drainage and formal debridement of skin and soft tissues. The authors observed that aspiration of more than 50 mL of fluid from Morel-Lavallée lesions was more frequent among lesions that recurred (83 %) as compared to those that resolved (33 %). Therefore, it has been recommend that aspiration of more than 50 mL of fluid from a Morel-Lavallée lesion should prompt operative intervention [20]. However, data of Morel-Lavallée lesions were not documented in the present series. Although, we did not quantify the initial drained amount of fluid in simple drainages, recurrent collection was observed in patients with initial copious drainage. Such patients should undergo repeated drainages and ultimately required proper debridement. The mortality rate was higher in closed type of degloving injuries. Notably, severe associated injuries such as traumatic brain injury and pelvic fracture were predominant in fatal cases which in fact were the contributing factors for increased mortality in closed DSTI. In addition, severe associated injuries may also lead to increased hospital length of stay.

The retrospective nature of the present study is one limitation. Detailed intervention and management of specific anatomical injuries were not well elaborated and the exact volume and the amount of fluid in the degloving injuries were available only for 37 cases based on computed tomography findings. Moreover, despite 11 cases with pelvic fracture had closed DSTI, Morel-Lavallée lesions were not documented which could be due to delayed diagnosis secondary to possible inconsistent clinical presentation. Lastly, this study lacks the exact details of the radiological investigation particularly for closed DSTI as the initial diagnose was primarily based on the clinical assessment. The tissue viability of the open/closed degloving injury, which is supposed to be a key factor relating to morbidity, mortality and ultimate result, was lacking in the available registry data and need further prospective work to be addressed. The time frame of management was not given in the database.

## Conclusions

Diagnosis of degloving injury is a challenge as initially the emphasis concentrates on the most urgent life and limb threatening issues. Also the fact that some of these injuries are initially subtle and tend to deteriorate over time to become obvious as swelling or skin changes and for that some lesions can be missed and diagnosed at late stage. Although modern imaging like CT and MRI can pick these injuries early, they are not asked for that particular indication and it is commonly observed that the radiologic report of these images underestimate or not properly comment on these injuries which are considered as less important incidental injuries of the subcutaneous tissue and unless we change our stand and start to think of it ahead, document and communicate proactively with the radiologist and multidisciplinary treating teams; the same challenges won’t be fixed. Early diagnosis and on-time management of degloving injury depend on a high index of suspicion, clear protocols and guidelines on the approaches of management, standardized diagnostic criteria, and more reliance on clinical guidance of imaging technology. Current evidence support the use of MRI to diagnose, characterize and guide treatment and follow up, whereas ultrasound tends to be useful at later stage and or for follow up.

The incidence of DSTI is around 4 % with greater predilection to males and young patients in our series. Three quarter of the cases is traffic-related injuries. DSTI injuries are mostly underestimated lesions, with higher association of morbidity and mortality, if mismanaged. Open DSTI are more likely to be associated with head and scalp region whereas closed type are evident in lower extremity injuries and pelvic fractures. A high index of suspicion is crucial for the diagnosis and management of closed DSTI as it needs a multidisciplinary tailored approach. Moreover, the lower incidence of skin complication could probably attribute to the early interventions. To provide appropriate care for these patients, early tissue restoration and effective rehabilitation are crucial. Still, the treatment guidelines for DSTI are not well established; so further studies are needed to resolve controversial issues for DSTI grading and optimal diagnostic and treatment approaches guided by evidence-based practice.

## Abbreviations

CT scanning: Computerized Tomography
DSTI: Degloving soft tissue injuries
MVC: Motor vehicle crash
VAC: Vacuum assisted closure
